# Prevalence of indication for lateral approach sinus floor augmentation in maxillary molar tooth deficiency: a retrospective study

**DOI:** 10.1038/s41598-026-55114-7

**Published:** 2026-05-25

**Authors:** Zülfiye Doğan, Arzum Güler Doğru

**Affiliations:** Department of Periodontology, Dicle University, Diyarbakır, Turkey

**Keywords:** Alveolar ridge, Cone beam computed tomography, Jaw edentulous, Maxillary sinus, Sinus floor augmentation, Anatomy, Diseases, Health care, Medical research

## Abstract

This retrospective study aimed to evaluate the frequency of lateral approach sinus floor augmentation (LASFA) in patients missing at least one maxillary molar, based on assessments of residual vertical bone height (RVBH) and maxillary sinus anatomy using cone-beam computed tomography (CBCT). A total of 402 CBCT scans meeting the inclusion criteria were analyzed, encompassing 458 maxillary sinuses. The RVBH was measured on ten equidistant mesiodistal slices beneath the maxillary sinus, and the mean value was recorded as the RVBH for each site. Patients were divided equally by sex and into four age groups (18–28, 29–39, 40–50, and > 50 years). Those with RVBH ≤ 5 mm were classified as requiring LASFA, and those with RVBH > 5 mm as not requiring the procedure. LASFA was indicated in 28.2% of patients, whereas 71.8% did not require it. The mean RVBH was 7.68 ± 3.69 mm (range: 1.31–19.73 mm). Age distribution showed no significant difference between groups (*p* = 0.714), whereas gender distribution did (*p* = 0.017), with a higher proportion of males requiring LASFA (58.9%) compared with females (41.1%). Within the study limitations, a substantial proportion of patients with maxillary molar tooth deficiency required LASFA. While RVBH exhibited a slight downward trend with advancing age, it was found to be higher in males than in females. These findings highlight the critical importance of pre-operative RVBH assessment via CBCT to ensure precise surgical planning.

**Supplementary Information:**

The online version contains supplementary material available at 10.1038/s41598-026-55114-7.

## Introduction

Deficiencies in oral and maxillary-facial functions are not limited to impaired chewing and phonation, but also cause maxillary and mandibular bone resorption. The replacement of missing teeth has an important functional and psychosocial effect^1,2^. Tooth loss leads to chronic alveolar ridge resorption and structural remodeling due to reduced mechanical stimulation and physiological stress^3,4^. In the posterior maxilla, this process is often exacerbated by maxillary sinus pneumatization, which significantly compromises the residual vertical bone height (RVBH) required for stable dental implant placement^5^. Given the anatomical proximity of the maxillary sinus to the posterior teeth, a meticulous pre-operative three-dimensional (3D) evaluation of the sinus floor is essential to ensure predictable clinical outcomes in implant-supported rehabilitation^6^.

Especially in the posterior maxilla, a considerable proportion of edentulous patients may require bone augmentation procedures due to advanced alveolar bone resorption to facilitate proper implant placement and to achieve predictable and satisfactory clinical outcomes^7^.

Lateral approach sinus floor augmentation (LASFA) is a frequently preferred technique among various augmentation modalities, particularly in cases where the residual bone volume is significantly limited. Despite being associated with postoperative complications, high costs, and procedural complexity, its clinical success and predictability have been firmly established^7–10^. With the improvement of surgical techniques and equipment, the effect of anatomical factors and the choice of surgical approaches has been continuously updated=^11–14^.

In sinus floor augmentation operations, The RVBH is an important factor determining the surgical approach^15^. RVBH also plays a major role in implant survival rates^16^. According to some studies, a pre-implant bone height of less than 5 mm is associated with a decreased survival rate. These findings indicate that a higher success rate could be achieved with greater alveolar bone height^17,18^. In a comprehensive sinus augmentation classification, it was reported necessary to perform LASFA in patients with RVBH ≤ 5 mm^19–21^. When the residul crest height is at an insufficient level for the application of a dental implant, sinus floor augmentations performed to obtain a sufficient RVBH provide extremely successful and predictable outcomes^22^. The selection of the sinus augmentation technique is made in most studies according to the vertical height of the remaining bone, and indications vary from 2-stage lateral antrostomy when there are conditions of severely resorbed maxilla, to a less invasive osteotomy technique when there is only mild resorption^23,24^. When the classifications and recommendations to date are examined, LASFA can be seen to be preferred in severely atrophic crests where the RVBH is ≤ 5 mm^25,26^. In a study by Balaji SM, LASFA was found to be appropriate for patients with RVBH ≤ 5 mm^20^.

While conventional two-dimensional (2D) imaging methods such as panoramic radiography offer low radiation doses and accessibility, they possess significant limitations in evaluating RVBH due to geometric impairment and overlapping anatomic structures, necessitating the use of 3D imaging^27–29^.

Cone-beam computed tomography (CBCT) is an imaging method of great importance in the dentomaxillofacial region^30^. CBCT imaging before sinus floor augmentation allows a detailed examination of the operation field. With the determination of potential pathologies in three dimensions (3D) and reducing patient morbidity, it allows the surgeon to plan the operation virtually in a more sensitive manner^31^.

In the guidelines related to the use of diagnostic imaging in implant dentistry published by the European Osseointegration Society, it is stated that CBCT can be of potential benefit for successful implant treatment in cases where there is limited RVBH and/or the residual bone width is clinically borderline^32^. Previous studies have reported that CBCT is an excellent imaging method for reliable measurements of maxillary sinus dimensions^32^. CBCT has become a fundamental diagnostic tool in implant dentistry for surgical planning and bone quality evaluation, providing accurate measurements of maxillary sinus dimensions with lower radiation doses compared to CT^33–36^.

Although previous studies have established general criteria for LASFA based on RVBH, there is a lack of localized data regarding the prevalence and influence of demographic factors on these indications. Therefore, this study aims to retrospectively evaluate the frequency of indication for LASFA in patients with at least one maxillary molar tooth missing by evaluating the RVBH and the maxillary sinus using CBCT.

## Material and method

### Patient selection

**Fig. 1:**
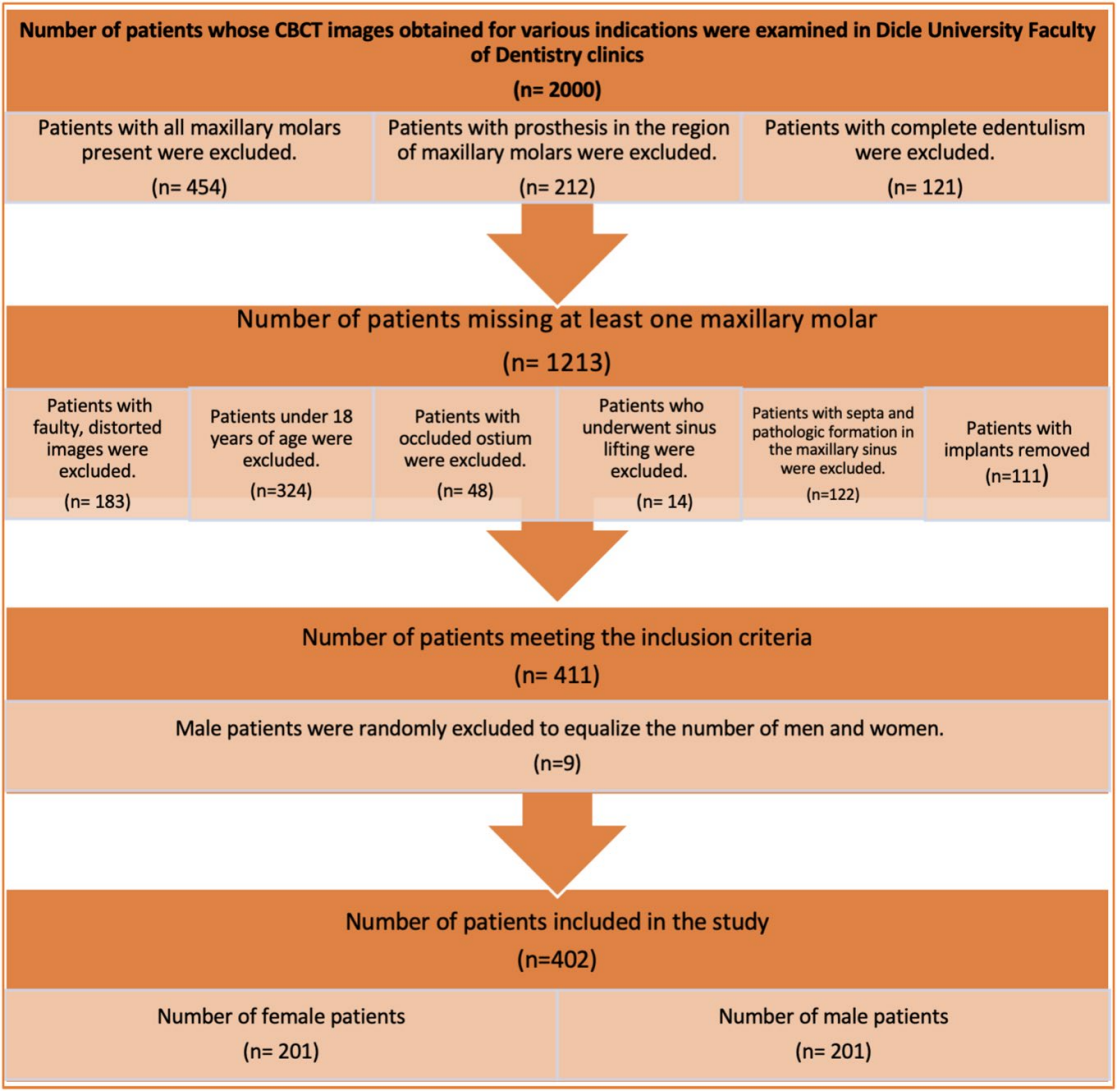
Flowchart illustrating the selection of the study sample. The diagram shows the initial number of CBCT scans assessed, the exclusion criteria applied, and the final number of patients and images included in the analysis.

A total of 402 patients who met the inclusion criteria were included. This sample provides sufficient power (95%) at a 5% significance level. The patient selection process and inclusion/exclusion criteria are summarized in Fig. 1. A retrospective examination was made of CBCT images obtained from patients with different indications who presented for any reason at the Dentistry Faculty Clinics of Dicle University between January 2014 and March 2024. From the CBCT images examined of 2000 patients, the images of 402 patients were found to be suitable for inclusion in the study; 201 males and 201 females. Measurements of the maxillary sinus were performed on 458 images; 229 from males and 229 from females. All the patients were in the age range of 18–79 years and had at least one maxillary molar tooth missing. The status of missing teeth in the right and/or left maxillary posterior region was evaluated separately in all the patients included in the study. Maxillary third molar teeth were disregarded.

CBCT images that conformed to the study inclusion criteria, taken from equal numbers of males and females, were used in the study.

### Patient groups and number

Patients were classified based on RVBH. Patients with an RVBH ≤ 5 mm were assigned to Group 1**,** indicating the need for LASFA, whereas patients with an RVBH > 5 mm were assigned to Group 2, in whom LASFAwas not indicated.

Both groups were further stratified according to sex (male/female) and age. Age was categorized into four groups: 18–28, 29–39, 40–50, and > 50 years**.** Comparisons were performed between groups for sex distribution and age categories.

### CBCT image acquisition and radiographic evaluation

CBCT images were obtained using a KaVo Dental i-Cat FLX 3D system (Brea, CA, USA) at the Department of Oral Diagnosis and Radiology, Faculty of Dentistry, Dicle University. Imaging parameters were set at 120 kVp, 5 mAs, 1.2 mm slice thickness, 8–9 s scanning time, and 0.3 mm voxel size**.** During image acquisition, patients’ heads were stabilized, and positioning was standardized by aligning the sagittal plane parallel to the device light guides and the Frankfurt plane parallel to the floor.

**Fig. 2:**
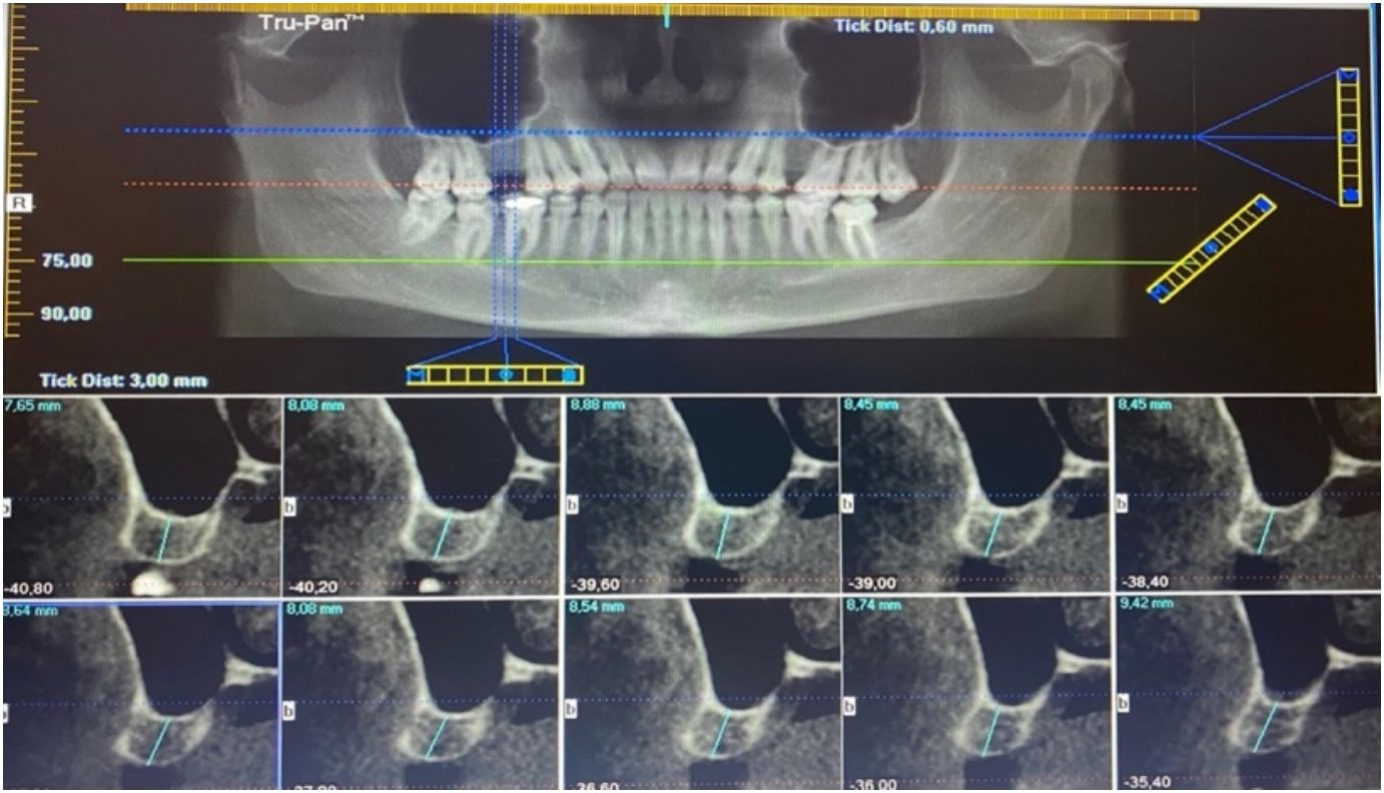
CBCT cross-sectional image demonstrating measurement of RVBH in an edentulous posterior maxilla. RVBH was measured as the vertical distance from the alveolar crest to the floor of the maxillary sinus on cross-sectional slices oriented perpendicular to the alveolar ridge.

All CBCT datasets were exported in DICOM format and analyzed using i-CAT Vision software (CT Dent Ltd., London, UK). Measurements were performed by a single examiner under standardized low-light conditions using the software’s measurement tools. Cross-sectional images were reconstructed by defining the dental arch on axial and coronal sections to generate panoramic views. RVBH was measured in edentulous areas beneath the maxillary sinus by averaging 10 cross-sectional slices taken at equal mesiodistal intervals. To ensure reproducibility, each measurement was performed at the midpoint of the buccolingual dimension and oriented perpendicular to the alveolar crest. The mean of these ten measurements was recorded as the final RVBH value for each site (Fig. 2). To assess intra-observer reliability, measurements were repeated in 10% of randomly selected cases after a one-week interval**.**

### Statistical analysis

A priori power analysis was performed using the findings of the referenced study and a small effect size value (w = 0.2). A total of 402 patients were included in this study, which provides a power level of 95% at a 5% type I error level**.** The conformity of continuous variables to normal distribution was examined by Shapiro–Wilk test. Continuous variables were expressed as mean ± standard deviation or median (minimum: maximum) and categorical variables were expressed as *n* (%). According to the normality test results, Mann Whitney U test was used for intergroup comparisons. Categorical variables were analyzed using Chi-square test. The Kruskal–Wallis test was employed to compare RVBH values among more than two independent age groups, as the data did not follow a normal distribution. Intra-observer reliability was assessed using the Intraclass Correlation Coefficient (ICC). SPSS (IBM Corp. Released 2012. IBM SPSS Statistics for Windows, Version 25.0. Armonk, NY: IBM Corp.) program was used.

## Results

To assess intra-observer reliability of the CBCT-based measurements, 10% of all measurements were randomly selected and re-measured by the same observer after a one-week interval. The intra-observer reliability analysis showed excellent agreement (ICC = 0.96; *p* < 0.001). Table 1 summarizes the distribution of patients based on their RVBH categories. Regarding the demographic profile of the study population, the majority of patients were aged over 50 years (44.50%). The gender distribution was equally split, with 50% male and 50% female participants. The overall mean RVBH for the entire study population was measured as 7.68 ± 3.69 mm (range: 1.31–19.73 mm). Based on the ≤ 5 mm threshold, LASFA was indicated in 28.20% (n = 129) of the cases, while 71.80% (n = 329) did not require the procedure. As shown in Table 1, patients in the LASFA-indicated group exhibited significantly lower mean RVBH values (3.52 ± 0.99 mm) compared to the non-indicated group (9.31 ± 3.01 mm).

**Table 1:** Distribution of patients by RVBH and its mean height.

Group	Number (n)	Percentage (%)	Mean RVBH ± SD	Median (mm)	Min.: Max (mm)
Patients with RVBH > 5 mm	329	71.80%	9.31 ± 3.01	8.59	5.08–19.73
Patients with RVBH ≤ 5 mm	129	28.20%	3.52 ± 0.99	4.13	1.31–5.00
Total Mean RVBH	458	100%	7.68 ± 3.69	7.32	1.31–19.73

The data are presented as mean ± standard deviation and median (minimum: maximum).

The distribution of LASFA indications and mean RVBH values according to age groups are detailed in Table 2. Statistical analysis revealed no significant difference in mean RVBH values across the different age groups (*p* = 0.202), and consequently, no post-hoc analysis was required. Furthermore, the proportion of patients requiring LASFA did not show a statistically significant variation based on age distribution (*p* = 0.714). Across all age categories, the mean RVBH values remained consistently lower in the LASFA-indicated groups compared to the non-indicated groups, with the oldest patient group (> 50 years) representing the largest proportion of the study population in both categories.

**Table 2:** Distribution of patients by age group according to RVBH categories.

Age group (years)	Patients with RVBH ≤ 5 mm			Patients with RVBH > 5 mm			*p*-value
	N (%)	Mean RVBH ± SD	Median (Min.: Max.)	N (%)	Mean RVBH ± SD	Median (Min.: Max.)	
18–28	11(8,50%)	3,65 ± 0,99 mm	4,01(1,85:4,79)	38(11,60%)	10,12 ± 2,95 mm	9,72(5,79:18,28)	0,202^a^
29–39	23(17,80%)	3,64 ± 1,02 mm	3,87(1,72:4,97)	61(18,50%)	10,04 ± 3,56 mm	9,66(5,35:19,73)	
40–50	33(25,60%)	3,54 ± 0,98 mm	3,62(1,57:4,89)	88(26,70%)	9,19 ± 2,69 mm	8,52(5,43:17,58)	
over 50	62(48,10%)	3,44 ± 1,01 mm	3,39(1,31:5)	142(43,20%)	8,85 ± 2,89 mm	8,26(5,08:17,30)	
Total	129			329			0,714^b^

RVBH = residual vertical bone height; values are presented as number of patients (n) and mean ± standard deviation. *p*-values indicate differences in age group distribution between RVBH ≤ 5 mm and RVBH > 5 mm groups, calculated using the Chi-square test for categorical counts (b). Mean RVBH comparisons were performed using Kruskal- Walli’s test (b) due to non-normal distribution.

Gender-based distribution of LASFA indications and corresponding mean RVBH values are presented in Table 3. No significant difference was observed in age distribution between the groups (*p* = 0.714). However, younger age groups (18–50 years) were less represented in the group requiring LASFA, whereas patients older than 50 years were more prevalent in this group. A statistically significant difference in gender distribution was observed between the groups (*p* = 0.017). The proportion of males was higher in the group requiring LASFA, whereas females were more prevalent in the group not requiring LASFA.

**Table 3:** Distribution of patients by gender according to RVBH categories.

Age group (years)	Patients with RVBH ≤ 5 mm			Patients with RVBH > 5 mm			*p*-value
	N (%)	Mean RVBH ± SD	Median (Min.: Max.)	N (%)	Mean RVBH ± SD	Median (Min.: Max.)	
Male	76(58,90%)	3,37 ± 1,02 mm	3,44(1,31:5,0)	153(46,50%)	9,26 ± 3,21 mm	8,71(5,08:19,73)	0,017^c^
Female	53(41,10%)	3,72 ± 0,93 mm	3,87(1,63:4,99)	176(53,50%)	9,35 ± 2,84 mm	6,77(5,14:17,58)	
Total	129(100%)			329(100%)			

RVBH values are presented as number of patients (n) and mean ± standard deviation (SD). *p*-value indicates differences in gender distribution between RVBH ≤ 5 mm and RVBH > 5 mm groups, calculated using the Chi-square test (c). Mean RVBH comparisons were performed using Mann–Whitney U test due to non-normal distribution.

## Discussion

The widespread bone resorption in the majority of cases without maxillary teeth and sinus pneumatisation occurring after tooth extraction usually prevent the placement of implants without any additional surgical procedures such as sinus elevation and bone grafts^37^. LASFA is an effective and highly predictable surgical technique that provides adequate bone volume in the atrophic posterior maxilla, thereby enhancing implant stability and long-term success^38,39^.

Voxel size is a critical parameter influencing CBCT image quality. While some studies recommend smaller voxel sizes for greater accuracy, others report no significant differences within the range of 0.125–0.4 mm^40–43^. In the present study, a voxel size of 0.3 mm was used, consistent with these recommendations to avoid elongation. This retrospective study utilized existing CBCT images from the Dicle University Faculty of Dentistry archive; thus, no additional radiation dose was administered to the patients.

In the current study, patients with RVBH ≤ 5 mm were included in the group requiring LASFA (Group 1), and those with RVBH > 5 mm in the group not requiring LASFA (Group 2).

LASFA was determined to be required by 28.20% of the current study patients and not by 71.80%. From a scan of the literature it was seen that the current study is one of very few to have investigated the prevalence of LASFA indication. In a similar study, Seong et al. examined 502 implant regions in the posterior maxilla and found that 54.2% were performed with a sinus augmentation procedure^44^. The results of the current study were not consistent with this as sinus floor augmentations to be performed with the transcrestal method were not included.

Mateo et al. reported a 49.38% requirement for LASFA and 29.63% for the transcrestal method in the posterior maxilla. While the current study used a ≤ 5 mm cutoff for LASFA, Mateo et al. included patients in the 5–6 mm range, highlighting the variability in indication thresholds. This comparison suggests that the ≤ 5 mm threshold should not be viewed as an absolute criterion, as alternative approaches may be considered depending on the specific clinical cutoff used in different methodologies^45^.

The mean RVBH measurement of the current study patients was found to be 7.68 ± 3.69 mm (min:1.31, max:19.73). In a study of 250 CBCT images, Padhye et al. examined 349 endentulous posterior maxilla localisations and reported the mean RVBH to be 7.37 ± 4.37 mm^46^. This value was consistent with and parallel to the current study values.

The mean RVBH measurement was found to be 3.52 ± 0.99 mm in the current study patients requiring LASFA, and 9.31 ± 3.01 mm in the group not requiring LASFA. From a scan of the literature, it was seen that this RVBH value of the group requiring LASFA was one of the lowest reported in the literature. The RVBH in patients requiring LASFA was found to be 3.69 ± 0.98 in a study by Hung et al.^47^ 3.60 ± 1.28 mm by Rosano et al.^48^ and 3.55 ± 1.37 by Aygün^49^. The parallel values of all these studies support the findings of the current study.

When examined according to the age groups of the current study patients requiring LASFA, the mean RVBH was found to be 3.65 ± 0.99 mm for the 18–28 years age group, 3.64 ± 1.02 mm for the 29–39 years group, 3.54 ± 0.98 mm for the 40–50 years group, and 3.44 ± 1.01 mm for the > 50 years group. These values for the age groups of the patients not requiring LASFA were 10.12 ± 2.95 mm, 10.04 ± 3.56 mm, 9.19 ± 2.69 mm, and 8.85 ± 2.89, respectively. In both groups it was seen that the mean RVBH value decreased as age increased.

No statistically significant difference was determined between the groups requiring LASFA or not in respect of the distribution of age groups (*p* = 0.714). The percentages of patients requiring and not requiring LASFA were 8.50% and 11.60% respectively in the 18–28 years age group, 17.80% and 18.50% in the 29–39 years group, 25.60% and 26.70% in the 40–50 years group, and 48.10% and 43.20% in the > 50 years age group. Although there was no statistically significant difference, it was seen that both the RVBH decreased and the need for LASFA increased together with ageing. In parallel with the current study findings, the results of a thesis study by Bütün Ö.E. also demonstrated no statistically significant difference in respect of age distribution when the need for LASFA was examined^50^.

Similarly, the RVBH in the posterior maxilla region was reported not to be affected by the age factor in studies by Liang et al. and Güler et al. Those studies emphasized that the main reasons could be the duration of being without teeth and prosthesis use^51,52^.

Kreisler et al. and Sharan and Madjar reported that the most important factor determining the RVBH is the time since the tooth was extracted^53,54^. Although the data of the current study are similar, as this was a retrospective study, this condition could not be evaluated, so information on this subject is limted.

Tang et al. found a difference between age groups in respect of alveolar bone height and stated that there was a decrease in RVBH together with ageing^55^.

When the gender distribution of the patients was examined in the groups requiring and not requiring LASFA, the RVBH value was found to be higher in females than in males in both groups. In the light of this finding, it can be said that there is greater resorption in males than in females.

In the thesis study of Aygün H., the median RVBH value was found to be 4.00 mm in females and 3.54 mm in males in a groups requiring LASFA^49^. Bu değerler bizim çalışmamızla paraleldir.

Although not fully understood, it has long been observed that there is a difference between the genders in respect of sensitivity to, development, and progression of bacterial diseases. In a study by Valerio M. et al., the concept of gender dimorphism as a response to pathogenic infection is supported, and it was emphasized that osteoclastic activity was increased in males compared to females, and therefore, bone resorption is greater in males^56^. In a study of rats by McAbee et al., it was seen that male rats showed greater bone loss and inflammatory infiltration than female rats^57^. In contrast, Wagner F. et al. and Kovavic I. et al. found no difference in bone resorption between the genders^58,59^. Studies by Xie Q et al. and Beat et al. reported greater resorption in females than in males^60,61^.

When comparisons were made of the current study patients in the groups requiring and not requiring LASFA according to gender, a statistically significant difference was observed according to gender distribution (*p* = 0.017). The rate of 53.50% females in the group not requiring LASFA was higher than the rate of 41.10% females in the group requiring LASFA. Conversely, the rate of 58.90% males in the group requiring LASFA was higher than the rate of 46.50% in the group not requiring LASFA (*p* = 0.017). The RVBH was found to be higher in females than in males, and the need for LASFA was significantly greater in males than in females.

Acharya A. et al. examined the CBCT images of 457 patients, and found a difference between the genders in respect of the requirement for sinus floor augmentation, consistent with the current study findings^62^. Similarly, Seong et al. also reported a gender difference in terms of sinus floor augmentation reequirement^44^.

The lack of statistical significance in LASFA indication prevalence across age groups may be attributed to the time elapsed since tooth extraction being the primary factor for resorption, rather than chronological age. Regarding gender, the significantly higher prevalence of LASFA indication in males compared to females (*p* = 0.017) may be associated with increased osteoclastic activity and greater alveolar bone resorption in males. Additionally, gender-specific variations in susceptibility to bacterial diseases and biological responses to their effects may contribute to these findings.

However, this was a retrospective study, so various factors such as the time since tooth extraction, prosthesis use, and systemic diseases could not be evaluated. Moreover, the study relied solely on CBCT measurements and a single RVBH cutoff to determine the indication for LASFA, which may not fully reflect the variability reported in other studies. Considering that the time since tooth extraction and other clinical factors may influence treatment decisions, further prospective studies are needed to examine these variables and validate the RVBH threshold.

## Conclusion

In this retrospective CBCT study, a substantial proportion of patients with missing maxillary molars were found to require lateral approach sinus floor augmentation, reflecting the prevalence of LASFA indication in this population. RVBH decreased slightly with age, and males had significantly higher RVBH values than females. These findings emphasize the importance of assessing RVBH for surgical planning and may help guide clinical decision-making regarding LASFA.

## Supplementary Information

Supplementary Information.

## Data Availability

The datasets generated and/or analysed during the current study are available as supplementary information files.
